# Patient-level factors are more salient than a legislation prohibiting minors in bars in predicting unintentional injury hospitalizations

**DOI:** 10.1186/s12889-019-7327-7

**Published:** 2019-07-29

**Authors:** Imelda K. Moise, Evan de Joya, Vinicius Okada Silva, Vanji Moise, Didi Bertrand Farmer, Adelisa Orantia

**Affiliations:** 1 Department of Geography, University of Miami, 1300 Campo Sano Ave, Coral Gables, FL 33124 USA; 2 Case Western Reserve University, 10900 Euclid Ave, Cleveland, OH 44106 USA; 3 Eastern University, 1300 Eagle Road, St Davids, PA 19087 UK; 4 Partners In Health, 800 Boylston Street, Boston, MA 02199 USA; 5 Illinois Department of Public Health, Division of EMS and Highway Safety, Springfield, IL 62701 USA

**Keywords:** Alcohol policy, Young people, Substance use, Alcohol, Prevention, Accidents, Motor vehicle

## Abstract

**Background:**

Alcohol related homicide, suicide and aggravated assault represent the largest costs for the state of Illinois. Previous research has examined the impact of some alcohol-related policies on youth alcohol use and alcohol-related harm in the United States but findings have been mixed. To our knowledge, no study has provided a detailed epidemiology of the relationship between the impacts of alcohol policies on unintentional injury in Illinois. Therefore, the purpose of this study is to determine whether a legislation that prohibit minors under 21 years old in establishments that serve alcohol is more salient than individual level factors in predicting hospitalization for traumatic unintentional injuries.

**Methods:**

A retrospective observational study of data abstracted from 6,139 patients aged 10 to 19 hospitalized in Illinois Level I and Level II trauma centers. Patient data from 2006 to 2015 was linked with the city-level alcohol-related legislation (*n* = 514 cities). The response variable was whether a patient tested positive or negative for blood alcohol concentration (BAC) at the time of admission. Mixed-effects logistic regression analyses were conducted to model the patient and city level legislation effect of having a positive BAC test result on hospitalizations after adjusting for the legislation and patient factors.

**Results:**

After adjustment, patients aged 15 to 19 and white patients who tested positive for BAC at the time of admission had the greater odds of hospitalization for traumatic alcohol-related unintentional injuries compared to patients who had a negative BAC test result. However, odds of hospitalization decreased for female patients and for those with private insurance, and over time, but a significant decrease in such hospitalizations occurred during 2010, 2014 and 2015. The alcohol-related legislation of interest was not a significant predictor of traumatic alcohol-related unintentional injury hospitalization.

**Conclusions:**

Patient-level covariates were significant predictors of traumatic alcohol-related unintentional injury hospitalization; an alcohol-related legislation may not reduce hospitalizations for young patients aged 10 to 19. Therefore, to prevent underage drinking and consequences, interventions should target sex/gender, race/ethnicity and focus on both individual and environmental strategies.

**Electronic supplementary material:**

The online version of this article (10.1186/s12889-019-7327-7) contains supplementary material, which is available to authorized users.

## Background

The Surgeon General of the United States (US)‘s 2007 call to action to prevent and reduce underage drinking serves as a reminder that underage drinking consequences extend beyond the health of the young person, with implications on society and the economy [1, 2], demonstrating the importance of prevention [3]. In the US, acute outcomes of underage drinking have been linked to the three leading causes of death among young people (homicide, suicide and unintentional injury) [2], with the severity of unintentional injuries increasing with alcohol use [4, 5]. For instance, in 2016, alcohol misuse accounted for 78.8% of all underage alcohol misuse–related emergency department visits, with young patients also more likely to be treated for an injury amid the visit and being hospitalized, suggesting the injury severity [6]. The mortality rate for this age group was particularly high among trauma young patients who tested positive for alcohol at the time of hospitalization [7]. Despite this fact and to the best of our knowledge, the link between young persons’ alcohol-related hospitalization and alcohol legislation has not been previously been investigated in Illinois.

Previous research has established that alcohol policies, such as a reduction in alcohol density, reduces alcohol use and related consequences by altering the physical access to alcohol [8–11]; but findings in the US have been mixed, in particular, the association between alcohol outlet density (on- and off-premises) and unintentional injuries (e.g., motor-vehicle crashes) [12, 13]. There is also a growing body of literature that recognizes the impact of some alcohol-related policies, youth alcohol use, and alcohol-related harm [14–16], however, much of the research up to now has primarily focused on alcohol outlet density and alcohol-related motor-vehicle crashes [9, 17–19]. In most of these studies “single vehicle nighttime crashes are widely used to indicate motor-vehicle crashes due to drinking and driving” [8]. However, whether this type of association exists in cohorts of young patients who test positive or negative for BAC at the time of admission at trauma centers in Illinois is unclear. We also know little about the current prevalence of traumatic alcohol-related unintentional injuries among young people, and/or how these outcomes have changed over time.

Individual risk factors for alcohol-related hospitalizations among young people include prior alcohol admission [20], inept parental monitoring, parent-child conflict, peer deviance, academic failure, sex/gender, and age [21, 22]. Recent work has established that hospitalization due to injuries are likely to vary across space, such that high and low levels of hospitalizations are concentrated in specific geographic areas [23, 24]. Additionally, the extent to which alcohol policies and individual-level risk factors are associated with alcohol-related hospitalizations among young people is likely to vary across geographic regions.

Therefore, there is potential to use city-level legislation/policy data and fine grained trauma center hospitalization data to better understand hospitalizations for unintentional injuries among young people, and variation in the strength of alcohol-related policies and individual factors [25]. Further, because trauma centers exist to treat the most serious, and often the most costly injuries, trauma registry data provide insight into the nature and extent of underage traumatic alcohol-related unintentional injuries [26]. Data from trauma centers can play an important role in monitoring the effectiveness of alcohol policies on unintentional injuries among young people. Additionally, analysis of trends and characteristics of those hospitalized can inform the design and deployment of tailored alcohol interventions, potentially enhancing their efficacy [20, 25].

The purpose of this study was 2-fold. First, we described the rates and risk of hospitalization among patients aged 10 to 14 and 15 to 19 due to traumatic unintentional injuries over time for both patients with positive and negative BAC test results at time of admission. Second, we determined the unique legislation and patient-level association between BAC levels and traumatic unintentional injury hospitalizations. We tested the hypothesis that if a city has a legislation that prohibit minors under 21 from entering any establishment licensed to sell alcoholic beverage, then we can expect a reduction in alcohol-related traumatic unintentional injury hospitalizations among adolescents.

## Methods

### Study design

This study was designed as a retrospective observational study involving patients aged 10 to 19 with and without positive blood alcohol concentration (cases and controls, respectively) over a 10-year period. Inclusion criteria were all of young patients aged 10 to 19 identified in the Illinois State Trauma Registry (ISTR) following presentation to level I and level II trauma centers. ISTR is a mandatory reporting database maintained by the Illinois Department of Public Health containing information about all traumas. This database is de-identified with respect to name and hospital, but includes patient demographic information, such as home address, gender, age, race, physiological data, mortality and discharge outcomes, and incident/scene address information. Cases were young patient encounters with a positive whole BAC test result at the time of admission, whereas controls were the young patients with a negative whole BAC test result at the time of admission. Only patients with complete records were used in the analysis. In addition, because we sought to determine whether a legislation that prohibit minors under 21 years old in establishments that serve alcohol is more salient than individual level factors in predicting traumatic unintentional injury hospitalization, we obtained city-level survey data of local liquor control authorities from the Illinois Liquor Control Commission (ILCC) conducted in 2013. Patient data (*n* = 6,139, patients with complete records) were then linked with the city-level alcohol-related legislation (*n* = 514 cities).

### Study settings

Illinois is no exception to the social burden of underage drinking consequences: alcohol related homicide, suicide, and aggravated assault represent the largest costs for the state, and when compared to other states in the US, “the harm from underage drinking averages $1,439 per youth” [27]. In 2012, young people ages 12 to 20 accounted for 9% of all alcohol-abuse treatment hospitalizations in the state. For this reason, Illinois has made prevention of underage drinking a top public health priority, and has implemented laws and penalties for driving under the influence (DUI), for underage drinking [28], and evidence-based strategies for reducing underage drinking. These include but not limited to the establishment of minimum drinking age (effective January 1, 1980), and the Illinois Zero Tolerance legislation that make it illegal for those under the age of 21 to drink and drive (Effective January 1, 1995). In addition, limiting the availability of alcohol (social, economic and physical) to young people through policies (e.g., a Class A misdemeanor to parents or guardians who knowingly permit their residence to be used by those under age 21) [28]. The adoption of a national minimum drinking age of 21 for “purchase or public possession” of alcohol [29], with strategies implemented via a comprehensive prevention approach. However, in Illinois, the detailed epidemiology of the relationship between the impacts of alcohol policies on unintentional injury has not been fully described.

### Measures

Patient demographic information, including the patient’s home address, age, sex, insurance status, race/ethnicity, admit/discharge date, external causes of injury (e-codes), were abstracted from ISTR. We abstracted patient information for all children (aged 10 to14) and adolescents (aged 15 to 19) admitted to any of the eleven Level I and Level II Trauma Centers in Illinois within linked cities (*n* = 514) with a related alcohol legislation of interest and without missing race, insurance and legislation information. Abstracted data were from between January 2006 and December 2015. The response variable was whether the young patient tested positive or negative for BAC at the time of admission, with BAC tests performed on a patient within 24 h after first trauma center encounter.

We focused on unintentional injuries, defined as those not resulting from self-harm or suicide attempts as used in previous studies [30, 31]. In Illinois, each hospitalization at a trauma center is given an external cause of injury (E-Code) based on the *International Classification of Diseases*, 9th Revision, Clinical Modification (ICD-9-CM), which is used to distinguish between intentional and unintentional injuries (Additional file 1).

Patient level covariates were selected based on previous literature and theory [15, 20]. Before analysis, the decision was made to classify race into white versus minorities (Chinese, Japanese, Hawaiian, Filipino, Korean, Asia Indian, Vietnamese, Samoan, Black or African American, American Indian, Alaska Native and Aleut). Patients who reported self-pay or government-subsidized programs, including Medicaid/Medicare were considered to have “no private insurance,” with those whose likely source of payment included any kind of private (e.g., HMO/PPO/POS) or commercial insurance reported as having “private insurance.” Patients whose method of payment was listed, as other, unknown, not billed for any reason, was empty or not applicable were reported as “other” insurance.

**Fig. 1:**
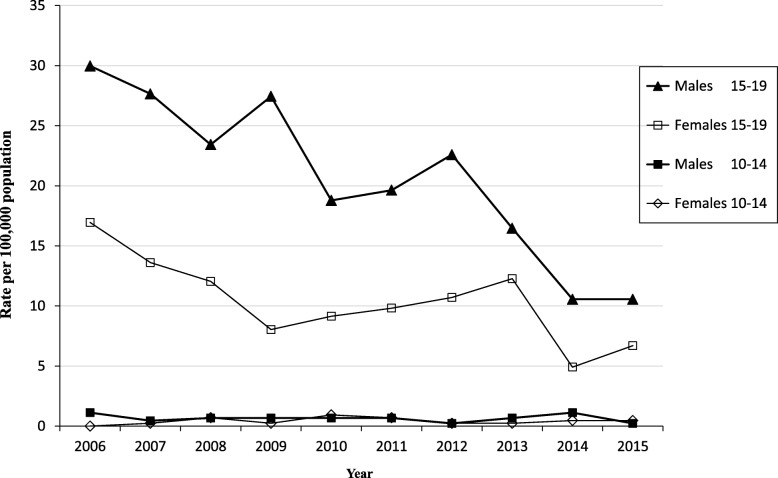
Flow chart indicating data processing steps for deriving the final study population of 6,139 patients aged 10 to 19 hospitalized at 11 Level I or Level II trauma centers in Illinois, 2006–2015

A city-level alcohol-related legislation used in the current study was obtained from the Illinois Liquor Control Commission (ILCC)‘s survey of local liquor ordinances conducted since August 22, 2013, with the data updated as of July 15, 2015. Among other questions, the survey asked city representatives to respond to a question that asked 1) “are minors under 21 years old allowed in bars and taverns”, with yes or no response options. The legislation was selected because it has been more widely adopted by 57.4% of cities that participated in the survey (*n* = 514 of 895 cities) compared to those often used in previous studies (e.g., “dry” towns, on-premises sale or consumption of liquor in bars, restaurants, and banquet halls) [8]. We then linked the legislation information to patient information by using “city name” as a unique identifier using Microsoft Access. For analysis, categorical variables were labeled as categorical for ease of interpretation in the mixed models. For example, variables city, county, zip code, BAC level, admission year and cause of injury was treated as nominal while variables under 21 allowed in bars or taverns, sex, insurance status and race were labeled as scale. Fig. 1 presents the data linkages and final study sample used in the current study of the patients aged 10 to 19, derived in stages.

### Statistical analysis

Descriptive and demographic data are presented as frequency and percent whereas the χ^2^ test was used to test for differences in patient level factors between young patients that tested positive for BAC and those that tested negative for BAC. Annual numbers, percentages and age-adjusted incidence rates of traumatic unintentional injuries (per 100,000 population) and 95% confidence intervals (CIs) were estimated (based on first encounter, excluding 0.38% repeat hospitalizations) and by age group, race and insurance status. Age-adjusted incidence rates were age-adjusted to the 2000 U.S. Standard Population (Census P25–1130) for all traumatic unintentional injuries; with crude rates for hospitalization calculated using the 2010 US Census (denominator population).

For the primary analyses, mixed-effects logistic regression models were estimated to evaluate the effect of an alcohol legislation on traumatic unintentional injury hospitalizations after adjusting for patient-level variables age, sex, insurance and race, and the interactions of legislation with age group. Interactions assessed whether the legislation may not be effective to a certain age group. The mixed-effects analysis were used to account for dependence resulting from patients being nested within cities. Predictors were added sequentially to the model, including BAC level, to evaluate the unique effect of BAC level after inclusion of the independent variables, in which the fixed effects can be interpreted as conditional on cities, with all random effects fixed at zero (e.g., unit-specific models). At the patient level, continuous predictors (age) was centered near their grand mean; binary predictors remained uncentered. Patient-level means for all predictors were included to represent contextual effects (e.g., the incremental effects of city characteristics after controlling for patient characteristics), all of which were retained regardless of statistical significance for proper interpretation of patient-level effects. Finally, the necessity for random slopes was evaluated separately for each patient-level predictor.

All models were estimated via Generalized Linear Mixed Model function in IBM SPSS Statistics, version 24.0, with binomial probability distribution, logit link and between–within denominator degrees of freedom. Improvement to model fit resulting from inclusion of random effects was evaluated via likelihood-ratio tests for nested models and Akaike Information Criterion (AIC) for non-nested models. Statistical significance of fixed effects was evaluated using *p* < .05.

## Results

In this cross-section retrospective study, data for 9,962 patients (of age 10 to 19 years) were abstracted from 2006 to 2015. Missing data were observed for approximately 23.3% (*n* = 1,880) of patients due to patients whose cities did not respond to the ILCC’s survey of local liquor ordinances. Two hundred and ninety patients were missing both insurance and race information, with an additional 930 patients missing only insurance information and 1,220 patients missing only race information while 45 patients were missing data for ≥1 model predictors. Final analyses were conducted on a sample of 6,139 patients aged 10 to 19 from 60.1% cities (*n* = 514 of 519 cities who responded to the legislation question of interest).

Of the hospitalized 6,139 patients, 81.9% (*n* = 5,063) tested negative for BAC while 17.4% (*n* = 1,076) tested positive for BAC at the time of admission. We found a statistically significant difference between BAC level and trauma center region, age group and race, *p* < .05 but not for insurance status and sex (Table 1). Significantly, among those aged 15 to 19, more males (64.4 and 66.2%) tested both positive and negative for BAC at the time of hospitalization than females (35.6 and 33.8%). The mean age was 16.8 years old.

**Table 1:** Demographic and trauma center region of adolescents aged 10–19 hospitalized by BAC level, Illinois, 2006–2015

Characteristic	Tested Negative for BAC (*n* = 5,063) n (%)	Tested Positive for BAC (*n* = 1,076) n (%)
Age group*		
10–14 years	810 (16.0)	32 (3.8)
15–19 years	4,253 (84.0)	1,044 (97.0)
Gender		
Male	3,261 (64.4)	712 (66.2)
Female	1,802 (35.6)	364 (33.8)
Insurance status		
No private insurance	1,914 (37.8)	434 (40.3)
Private Insurance	3,149 (62.2)	642 (59.7)
Race*		
Blacks and other minorities	1,082 (21.4)	171 (15.9)
Whites	3,981 (78.6)	905 (84.1)
EMS Trauma Center Region*		
Region 1	264 (5.2)	93 (8.6)
Region 2	980 (19.4)	181 (16.8)
Region 3	886 (17.5)	50 (4.6)
Region 4	145 (2.9)	38 (3.5)
Region 5	57 (1.1)	10 (0.9)
Region 6	173 (3.4)	61 (5.7)
Region 7	784 (15.5)	184 (17.1)
Region 8	572 (11.3)	130 (12.1)
Region 9	765 (15.1)	203 (18.9)
Region 10	199 (3.9)	50 (4.6)
Region 11	238 (4.7)	76 (7.1)

Chi-square test

*BAC* blood alcohol concentration

*EMS* emergence medical service

We conducted bivariate analyses using the X^2^ test for categorical variables

*P < .05

**Fig. 2:**
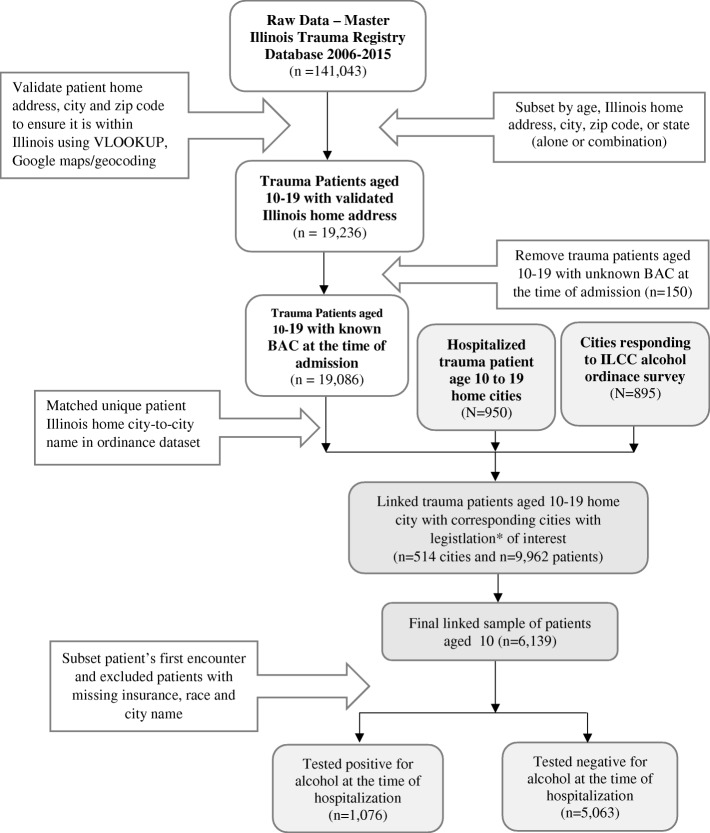
Variation in rates of alcohol-related traumatic unintentional injuries over time

Across all sex, age-subgroups and BAC level, rates of hospitalizations for traumatic unintentional injuries per 100,000 patients aged 10 to 19 decreased from 2006 to 2010, increased slightly during 2011 to 2012, and continued to decline during 2013 and 2014 before increasing slightly in 2015. However, when stratified by sex, age and BAC level, variations emerges. In particular, hospitalizations for traumatic unintentional injuries decreased by 9.5% (from 14.9% in 2006 to 9.4% in 2015) in young patients who tested positive for BAC at the time of admission but increased by 4.7% for young patients who tested negative for BAC at the time of admission. This is a significant outcome. In addition, although the rate of hospitalization fluctuated consistently over time for both sexes and age group, the rate increases with age for both males and females (Fig. 2). Notably, rates remained constantly low overtime for males and females aged 10 to 14.

As previously discussed, many studies have linked acute outcomes of underage drinking and unintentional injuries among young people. For example, from 2006 to 2015, an average of 115 patients were hospitalized due to alcohol-related unintentional injuries, for an annual age adjusted rate of 49.5 per 100,000 population. Nearly three-quarters (73.0%) of all alcohol-related traumatic unintentional injury hospitalizations occurred due to motor vehicles (785/1,076 unintentional injuries) while 11.8% (127 of 1,076 unintentional injuries) occurred due to falls (Table 2). The motor vehicle age adjusted rate for traumatic unintentional injury hospitalization was 27.5 per 100, 000 population for patients who tested negative for BAC and 6.3 per 100, 000 population for patients who tested positive for BAC at the time of admission. Most motor vehicle alcohol-related injuries were a result of motor vehicle traffic accident due to loss of control, without collision on the highway (14.6%, *n* = 115) or other motor vehicle traffic accident involving collision with motor vehicle injuring driver of motor vehicle (25.9%, *n* = 203).

**Table 2:** Average annual numbers and age-adjusted rates of traumatic unintentional injury hospitalizations by BAC test result and type of injury, Illinois, 2006–2015

	Negative BAC Test Result			Positive BAC Test Result		
Mechanism/case of injury^a^	*n*	%	Age-adjusted Rate^b^ (95% CI)	*n*	%	Age-adjusted Rate^b^ (95% CI)
Total	5,063	100.0	40.8 (0.815–0.824)	1,076	100.0	8.7 (0.166–0.185)
Chocking/suffocation	1	< 0.0	< 0.0	< 0	< 0.0	0.0
Cut/pierce	44	0.9	0.4 (0.006–0.011)	22	2.0	0.2 (0.014–0.031)
Drowning/submersion	7	< 0.1	< 0.1	1	< 0.1	< 0.0
Falls	488	9.6	3.9 (0.089–0.105)	127	11.8	1.0 (0.100–0.139)
Fire/burn	66	1.3	0.5 (0.010–0.017)	7	< 0.7	< 0.1
Firearm	97	1.9	0.8 (0.016–0.023)	11	1.0	0.1(0.005–0.018)
Machinery	17	0.3	0.1 (0.002–0.005)	1	< 0.1	< 0.0
Motor vehicle	3,410	67.4	27.5 (0.074–0.089)	785	73.0	6.3 90.703–0.757)
Natural/environment	14	0.3	0.1 (0.002–0.005)	7	< 0.7	< 0.1
Other*	42	0.8	0.3 (0.006–0.011)	6	< 0.6	< 0.0
Overexertion	3	< 0.1	< 0.0	0	< 0.0	< 0.0
Pedal cyclist, other	592	11.7	4.8 (0.108–0.126)	74	6.9	0.6 (0.055–0.086)
Poisoning	17	0.3	0.1(0.002–0.005)	10	0.9	0.1 (0.005–0.017)
Struck by/against	250	4.9	2.0 (0.044–0.056)	16	1.5	0.1(0.009–0.024)
Unspecified	15	0.3	0.1(0.001–0.005)	9	< 0.8	< 0.1

Source: Illinois Department of Health Trauma Registry

^a^Extrnal cause of injury is based on E-codes

^b^Age-adjusted hospitalization rates are based on age-specific hospitalization rates per 100,000 population in 10 to 19 age group. Age-adjusted hospitalization rates are computed by the direct method, using as the standard population the age distribution of the total population of the United States for the year 2000. Hospitalization records with missing race, insurance and legislation of interest nformation were excluded from the analysis

Abbreviations: CI, confidence interval; BAC, Blood Alcohol Concentration

*Other, not elsewhere classified

Intraclass correlations for all outcome and predictor variables were estimated using an empty model for each variable (e.g., random intercept only, no predictors). Approximately, only 7.8% of the variability in hospitalizations were due to between-city-differences. The probability of being hospitalized due to traumatic unintentional injuries across cities was 39.2%. Parameter estimates and implied effects from final models are shown in Table 3. All patient-level predictors were statistically significant, whereas a weak positive correlation (β = 0.097) was found between the city-level predictor (legislation) and alcohol-related unintentional injury hospitalizations (OR = 1.102; 95% CI, 0.889 to 1.367, *p =* 0.374). The relationship is not statistically significant, with a sample of 1,076 city-cases.

**Table 3:** Mixed-effects logistic regression analysis for traumatic unintentional injury hospitalizations for patients who tested postive for BAC at the time of hospitalization, Illinois, 2006–2015 (*n* = 1,079)

	Coefficient	Std. Error	t	*p*-value	Odds ratio	Confidence Interval (95%)
Intercept	−1.805	0.1662	−10.863	0.000	0.164	0.119–0.228
2015	−0.688	0.1706	−4.033	0.000	0.503	0.36–0.702
2014	−0.734	0.1778	−4.125	0.000	0.48	0.339–0.681
2013	−0.162	0.1542	−1.047	0.295	0.851	0.629–1.151
2012	−0.115	0.1483	−0.775	0.438	0.891	0.667–1.192
2011	−0.21	0.1458	−1.441	0.150	0.81	0.609–1.079
2010	−0.412	0.1494	−2.755	0.006	0.663	0.494–0.888
2009	−0.179	0.1422	−1.26	0.208	0.836	0.633–1.105
2008	−0.239	0.1414	−1.693	0.090	0.787	0.597–1.039
2007	−0.104	0.1353	−0.765	0.444	0.902	0.692–1.176
2006	0^b^	~	~	~	~	~
Age	0.405	0.0307	13.19	0.000	1.499	1.411–1.592
Gender	−0.072	0.0838	−0.861	0.389	0.93	0.79–1.096
Age*gender	−0.036	0.0518	−0.69	0.490	0.965	0.872–1.068
White	0.583	0.1154	5.056	0.000	1.792	1.429–2.246
Insurance	−0.275	0.0814	−3.372	0.001	0.76	0.648–0.891
Minors under 21^a^	0.097	0.1097	0.888	0.374	1.102	0.889–1.367
Minors under 21^a*^age	−0.106	0.0551	−1.929	0.054	0.899	0.807–1.002

Note:

Years indicate year of hospitalization

Of all the 1,243 cities in Illinois, over a 1000 cities responded to the Illinois Liquor Control Commission (ILCC) survey of local liquor-related ordinances. Of the 1000 cities that responded to the survey, 66.7 unique cities (*n* = 667) matched the home cities of young patients in this study. Of these cities, 514 responded to the question that asked whether minors under 21 years old are allowed in bars and taverns (yes vs. no). Cities whose responses were missing or unknown were excluded from the analysis

^a^Minors under 21 allowed in bars and taverns that sell liquor

^b^This coefficient is set to zero because it is redundant

Age* gender = interactions between age and gender

Minors under 21* age= interactions between minors under 21 and age

However, while no significant interaction were found between the patient’s age and sex, the unique effect of the city-level alcohol legislation differed significantly by a young patient’s age, as indicated by a statistically significant interaction. More specifically, cities with an alcohol–related legislation that prohibit minors under 21 from entering any establishment licensed to sell alcoholic beverage had 1.057 times less odds of having young patients aged 10 to 19 hospitalized for alcohol-related traumatic unintentional injuries, holding all fixed effects constant and random effects at zero. Being older (odds ratio (OR) =1.499; 95% CI, 1.411 to 1.592, *p* > 0.05) and being white (OR = 1.792; 95% CI, 0.1.429 to 2.246, *p* > 0.05) does increase the odds of being hospitalized for an alcohol-related traumatic unintentional injury. Having private insurance decreases the odds of hospitalization (OR = 0.76; 95% CI, 0.648 to 0.891, *p* > 0.05) and the odds of hospitalization decreased over time, but a significant decrease in such hospitalizations occurred during 2010, 2014 and 2015.

## Discussion

Our study demonstrated the high burden of traumatic unintentional injury hospitalizations among young people. Between 2006 and 2015, there was a steady decline in all traumatic unintentional injury hospitalization regardless of BAC test result for children 10 to 14 years and among adolescents aged 15 to 19, similar to previous studies [32–34]. A combination of environmental strategies such as public policies and implementation of evidence-based strategies at the community-level may have contributed to the decline.

Over time, we observed differences in rates of hospitalization by BAC level, in particular substantial decline (9.4%) in hospitalization for young patients who tested positive for BAC at the time of admission but an increase (4.7%) in young patients who tested negative at the time of admission between 2006 through 2015, a pattern that has been recognized [35]. Children age 10 to 14 years had a constant declining trend through the study period, likely related to age and developmental abilities and exposure to potential exposures [36, 37]. We also observed consistently higher rates of hospitalizations for traumatic unintentional injury among young male patients aged 15 to 19 and among white young patients. These results match those of a previous review that report racial and gender differences in unintentional injury rates [34] and suggests that alcohol use and drinking begin in late adolescence [38]. These trends are significant since they can be highly indicative as to what might be expected in the future, an effective approach to understanding alcohol-related traumatic unintentional injuries among young people.

An unanticipated finding was the slight increase in rates of females aged 15 to 19 in 2015. This finding, while preliminary, may suggest that current strategies are less effective in preventing the consumption of alcohol, and subsequent alcohol-related unintentional injury hospitalizations in this demographic group. These results are in line with those of previous studies [39–41] and while the observed increasing rates need to be monitored to establish their viability, this finding has important implications for developing sex/gender-informed interventions and prevention of related consequences to better target adolescent female drinkers.

Most of the alcohol-related unintentional injury hospitalizations were due to motor vehicles and falls. Motor vehicles and fall-related hospitalizations were seen in 68.3% of patients, followed by falls (10.0%), a proportion that was similar to others reported in the literature [34]. It appears that irrespective of motor vehicle and fall rates, the percentage of patients with consequent alcohol-related unintentional injuries remain relatively stable.

The intent of our multilevel analysis was to determine whether a unique legislation (minors under 21 are or not allowed in establishments that serve alcohol) and patient-level factors are associated with the odds of being hospitalized for alcohol-related traumatic unintentional injuries among young people ages 10 to 19. Contrary to our hypothesis, we found no correlation between traumatic alcohol-related unintentional injury hospitalizations and a city-level alcohol-related legislation. This finding is contrary to previous studies [16, 42], which linked alcohol bans on health outcomes, but they are broadly consistent with an earlier study that found negative results when evaluating the link between on- and off premises outlet density and fatal and non-fatal motor vehicle crashes. In a multicity study in California, McCarthy [43] found little effects of an alcohol-related legislation on driver-crashes. It is possible that other alcohol-related legislation and factors and/or their combination have indeed contributed to altering the physical access to alcohol to minors in Illinois and that to prevent underage drinking and consequences, interventions should focus on both individual and environmental strategies. This is an important issue for future research.

However, a negative significant interaction was found between the city-level alcohol-related legislation and alcohol-related traumatic unintentional injury hospitalization. There are several possible explanations for this result. It may be due to a limited sample size in young patients aged 10 to 19 who had positive BAC levels at the time of hospitalization due to unintentional injuries, suggesting that the legislation (minors under 21 are or not allowed in establishments that serve alcohol) may not be effective for reducing alcohol-related unintentional injury hospitalizations among certain age groups. It is also possible that a combination of other public policies and community-level evidence-based strategies such as those that limit the physical, social, and economic availability of alcohol to minors contributed to this finding. These include polices that make it illegal for drivers aged under 21 years to drive after drinking, providing mechanisms for early identification of problem drinkers [2].

This study is not without limitations. Due to its reliance on trauma center data, this could have resulted in underestimation of the unintentional injury hospitalizations as trauma centers exist to treat the most serious, and often the most costly injuries as well as the small number of young patients who tested positive for BAC at the time of admission. However, this is also a strength to this study, because we studied the hospitalizations and outcomes in children and in early adolescence, we expect trends in these high-risk groups to be indicative of overall trends. Second, excluded from the analysis are patient records with missing insurance, race and legislation information, which may lead to underestimation of the unintentional injury hospitalizations. Another limitation is in regards to generalizability. Illinois is unique in the comprehensiveness and maturity of its trauma system and the occurrence of traumatic unintentional injuries from this study may not be applicable to other communities. However, potential solutions to this problem could have local and national relevance.

## Conclusions

This study shows that both alcohol-related legislation and patient-level factors can contribute either positively or negatively to traumatic unintentional injury hospitalizations among young people. We demonstrate that both individual and environmental strategies are effective strategies for curbing the consequences of underage drinking. Given the observed increasing rates of alcohol-related traumatic unintentional injury hospitalizations among female and ethnic minority groups, sex/gender and race/ethnic targeted-interventions and continuous and sufficient funding for state and community level programs can have a critical impact on prevention of underage drinking at this critical transitional life stage. Although we did not find a significant relationship between the city-level alcohol-related legislation used in this study with traumatic unintentional injury hospitalizations, we did find a negative interaction between the city-level alcohol-related legislation and traumatic unintentional injury hospitalizations. An implication of this is the possibility that the legislation-- minors under 21 are or not allowed in establishments that serve alcohol may not be effective for reducing alcohol-related unintentional injury hospitalizations among certain age groups, in particular those under 19 years old. Future research should account for limitations presented in this study and incorporate other legislations, such as bans on alcohol and alcohol density.

## Additional file

Additional file 1: ICD-9-CM codes for external causes of injury for unintentional injury. (DOCX 13 kb)

## Data Availability

The datasets supporting the conclusions of this article are available upon reasonable request to the Illinois Department of Public Health following Institution Review Board Approval.

## Abbreviations

BAC: Blood alcohol concentration
IDPH: Illinois Department of Public Health
