# Correction to: Patient-level factors are more salient than a legislation prohibiting minors in bars in predicting unintentional injury hospitalizations

**DOI:** 10.1186/s12889-019-7448-z

**Published:** 2019-08-14

**Authors:** Imelda K. Moise, Evan de Joya, Vinicius Okada Silva, Vanji Moise, Didi Bertrand Farmer, Adelisa Orantia

**Affiliations:** 1 Department of Geography, University of Miami, 1300 Campo Sano Ave, Coral Gables, FL 33124 USA; 2 Case Western Reserve University, 10900 Euclid Ave, Cleveland, OH 44106 USA; 3 Eastern University, 1300 Eagle Road, St Davids, PA 19087 UK; 4 Partners In Health, 800 Boylston Street, Boston, MA 02199 USA; 5 Division of EMS and Highway Safety, Illinois Department of Public Health, Springfield, IL 62701 USA

**Correction to: BMC Public Health (2019) 19:1010**

**https://doi.org/10.1186/s12889-019-7327-7**

It was highlighted that in the original article [1] Fig. 1 and Fig. 2 were interchanged. This Correction article shows the correct Fig. 1 and Fig. 2. The original article has been updated.

**Fig. 1:**
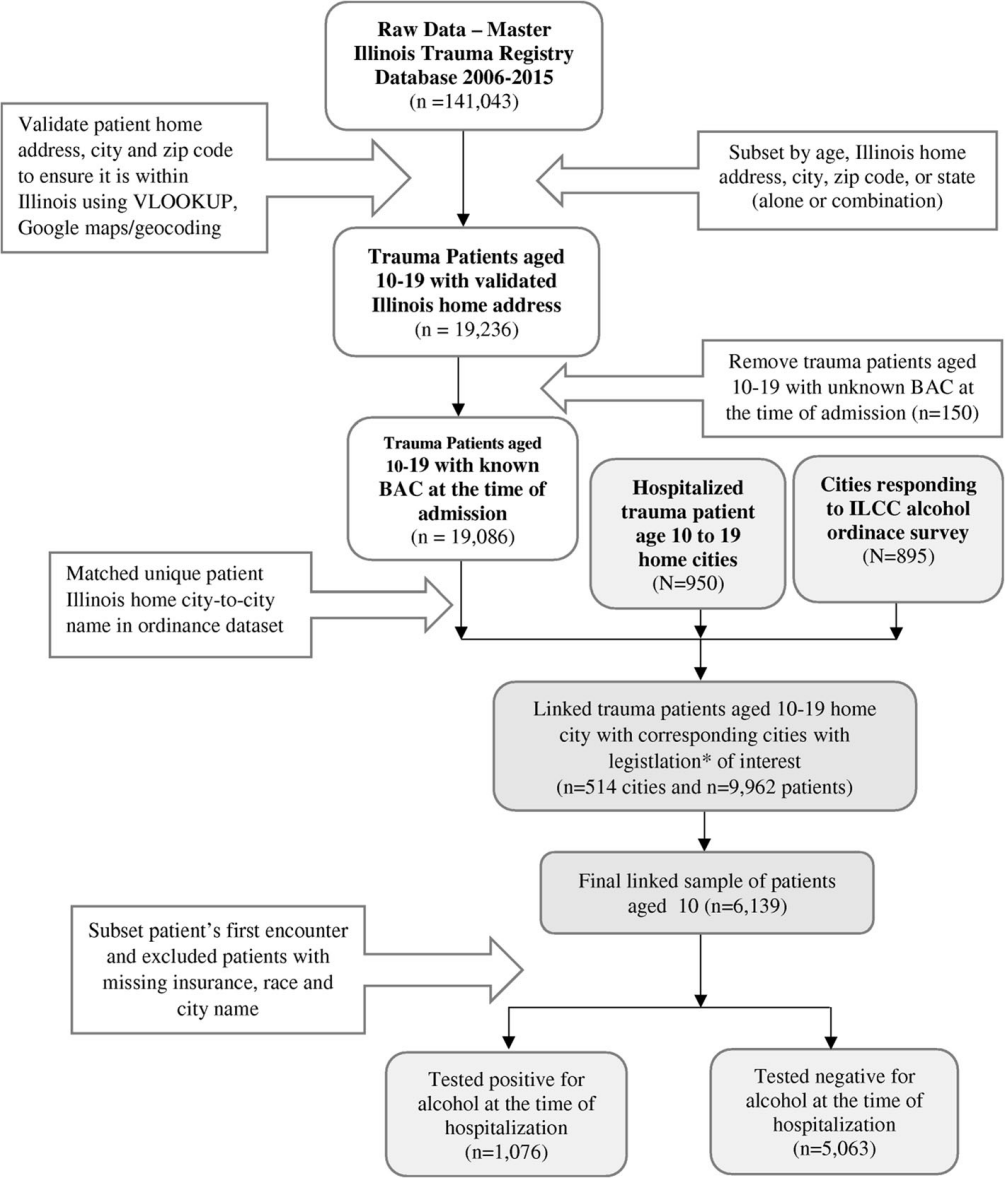
Flow chart indicating data processing steps for deriving the final study population of 6139 patients aged 10 to 19 hospitalized at 11 Level I or Level II trauma centers in Illinois, 2006–2015

**Fig. 2:**
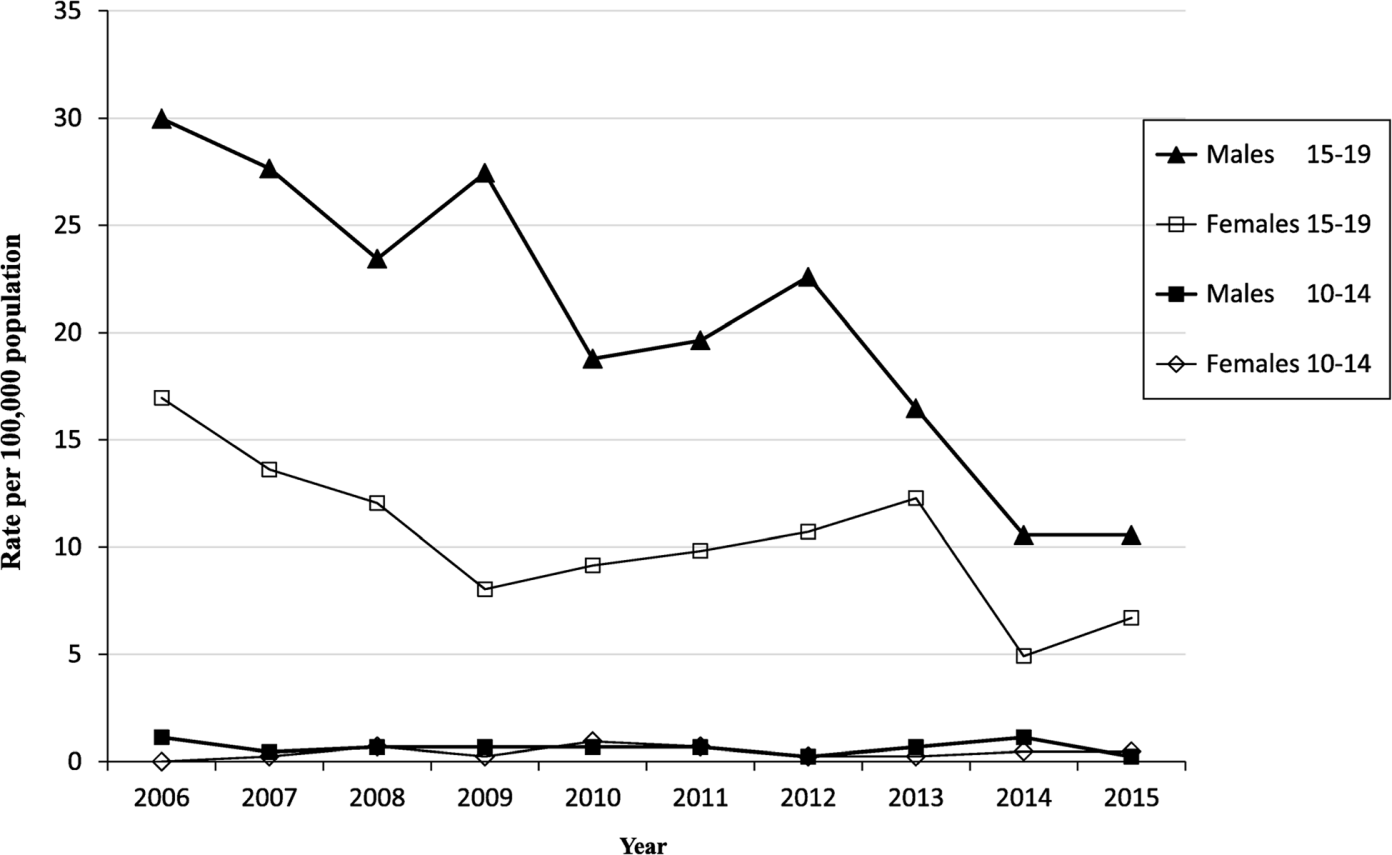
Variation in rates of alcohol-related traumatic unintentional injuries over time

## References

[1] Moise (2019). Patient-level factors are more salient than a legislation prohibiting minors in bars in predicting unintentional injury hospitalizations. BMC Public Health.

