# Supplementary material for: Patient-level factors are more salient than a legislation prohibiting minors in bars in predicting unintentional injury hospitalizations
Source: BMC Public Health. 2019 Jul 29;19:1010. doi: 10.1186/s12889-019-7327-7 (PMC6664708; doi:10.1186/s12889-019-7327-7)
Supplement: Supplementary file 1 — ICD-9-CM codes for external causes of injury for unintentional injury. (DOCX 13 kb) [file 12889_2019_7327_MOESM1_ESM.docx]

| Cause of injury | E-Code |
| --- | --- |
| All injury | E800-E869, E880-E929 |
| Cut/pierce | E920 (.0-.9) |
| Drowning/submersion | E830 (.0-.9), E832 (.0-.9), E910 (.0-.9) |
| Fall | E880.0-E886.9, E888 |
| Fire/burn | E890.0-E899, E924 (.0-.9), |
| Firearm | E922 (.0-.3, .8,.9) |
| Machinery | E919 (.0-.9) |
| Motor vehicle | E810-E819 (.0-.9) |
| Natural/environmental | E900.0-E909, E928 (.0-.9) |
| Overexertion | E927 |
| Poisoning | E850.0-E869.9 |
| Struck by, against | E916-E917.9 |
| Other and not elsewhere classified | E846-E848, E914-E915, E918, E921 (.0-.9), E922 (.4-.5), E923(.0-.9)E925.0-E926.9, E928 (.3, .8), E929 (.0-.5, .8) |
| Unspecified | E887, E928.9, E929.9 |

**Appendix A**

ICD-9-CM codes for external causes of injury for unintentional injury
